# Stromal Signals Dominate Gene Expression Signature Scores That Aim to Describe Cancer Cell–intrinsic Stemness or Mesenchymality Characteristics

**DOI:** 10.1158/2767-9764.CRC-23-0383

**Published:** 2024-02-23

**Authors:** Julian Kreis, Bogac Aybey, Felix Geist, Benedikt Brors, Eike Staub

**Affiliations:** 1 The healthcare business of Merck KGaA, Darmstadt, Germany.; 2 Faculty of Biosciences, Heidelberg University, Heidelberg, Germany.; 3 Division of Applied Bioinformatics, German Cancer Research Center (DKFZ), Heidelberg University, Heidelberg, Germany.; 4 German Cancer Consortium (DKTK), German Cancer Research Center (DKFZ), Heidelberg University, Heidelberg, Germany.; 5 Medical Faculty Heidelberg and Faculty of Biosciences, Heidelberg University, and National Center for Tumor Diseases (NCT), Heidelberg, Germany.

## Abstract

Epithelial-to-mesenchymal transition (EMT) in cancer cells confers migratory abilities, a crucial aspect in the metastasis of tumors that frequently leads to death. In multiple studies, authors proposed gene expression signatures for EMT, stemness, or mesenchymality of tumors based on bulk tumor expression profiling. However, recent studies suggested that noncancerous cells from the microenvironment or macroenvironment heavily influence such signature profiles. Here, we strengthen these findings by investigating 11 published and frequently referenced gene expression signatures that were proposed to describe EMT-related (EMT, mesenchymal, or stemness) characteristics in various cancer types. By analyses of bulk, single-cell, and pseudobulk expression data, we show that the cell type composition of a tumor sample frequently dominates scores of these EMT-related signatures. A comprehensive, integrated analysis of bulk RNA sequencing (RNA-seq) and single-cell RNA-seq data shows that stromal cells, most often fibroblasts, are the main drivers of EMT-related signature scores. We call attention to the risk of false conclusions about tumor properties when interpreting EMT-related signatures, especially in a clinical setting: high patient scores of EMT-related signatures or calls of “stemness subtypes” often result from low cancer cell content in tumor biopsies rather than cancer cell–specific stemness or mesenchymal/EMT characteristics.

**Significance:**

Cancer self-renewal and migratory abilities are often characterized via gene module expression profiles, also called EMT or stemness gene expression signatures. Using published clinical tumor samples, cancer cell lines, and single cancer cells, we highlight the dominating influence of noncancer cells in low cancer cell content biopsies on their scores. We caution on their application for low cancer cell content clinical cancer samples with the intent to assign such characteristics or subtypes.

## Introduction

The ability to switch from a stationary epithelial cell state to a motile mesenchymal cell state, called epithelial-to-mesenchymal transition (EMT), is essential for stem cell differentiation and dedifferentiation. This capability is a key driver of tumor cell plasticity for tumor cells, which is required for tumor cells to invade distant tissues and metastasize (1). Using bulk RNA sequencing (RNA-seq) data, multiple gene expression signatures have been postulated to describe stemness (2, 3), mesenchymality (4–7), and EMT (8–12) properties (hereafter referred to as EMT-related signatures) of cancer cells in colorectal, breast (BRCA), glioblastoma (GBM), and head and neck squamous cell carcinoma (HNSC). Subsequently, pioneering cancer characterization programs from The Cancer Genome Atlas (TCGA; ref. 13) have applied these signatures for cancer subtype identification or characterization of cancer-specific traits.

However, the expression profiles of bulk RNA-seq samples represent a complex mixture of pathway activity within cells in combination with heterogeneous cell type compositions. In samples with a high cancer cell content, expression signatures may provide high precision for cancer cell–intrinsic molecular states. In samples with comparably low cancer cell content, differences in cell composition between samples could dominate the scores of signatures originally intended to measure cancer cell–intrinsic properties. Multiple studies have highlighted the strong dependency of selected gene expression signatures on tumor microenvironment (TME) composition, limiting their applicability for cancer subtype stratification (14, 15), clinical study designs (16–18), and preclinical cancer models (12). Similarly, tumor and stromal cell expression profiles from microdissected and single-cell RNA-seq (scRNA-seq) samples from colorectal cancer (19–22), ovarian (23), HNSC (14), and pan-cancer (24) studies reemphasized such dependencies and identified fibroblasts as crucial contributors to the elevated scores of different mesenchymal subtype signatures. Some research groups have reinterpreted the influence of fibroblast content on distinct mesenchymal subtypes as a sign of fibrosis (20) and others as artifacts in the sampling procedure (14). Eide and colleagues argued that cancer cell–intrinsic processes control stromal composition and maintain the biased subtype by partly correcting for stromal contamination in colorectal cancer tumors (12). This conceptual problem is evident for several cancer indications; however, consensus on the experimental and computational procedures required for correctly assigning EMT-related tumor subtypes is lacking. In addition, a comprehensive analysis of a large panel of EMT/mesenchymality-related subtype signatures in the presence of varying cancer cell content in samples, or the assessment of expression of individual signature genes on the level of single cells, has not been undertaken.

Here, we dissected the influence of cells in the surrounding tumor tissue (i.e., the microenvironment and macroenvironment) with a focus on the influence of fibroblasts and immune cells on commonly used gene expression signatures for EMT-related phenotypes. To this end, we used RNA-seq data from both bulk tumor samples and single cells. Using RosettaSX, our published platform for gene expression signature scoring, and comprehensive analyses of scRNA-seq data, we investigated the association between EMT-related signature profiles and cancer cell/stroma content in samples of different tumor indications. We assessed the risk of premature conclusions when not considering the cell type composition of tumor samples in the assessment of EMT-related gene expression signatures, specifically for HNSC, BRCA, GBM, and colorectal cancer.

## Materials and Methods

### Data Preprocessing

RNA-seq count data of TCGA and Cancer Cell Line Encyclopedia (CCLE) consortia were normalized using the trimmed mean M-values (TMM) normalization of the edgeR (25) R-package using default parameters. Similarly, we complied with previously described methods to integrate pseudobulk and TCGA bulk samples (26). Briefly, we normalized bulk RNA counts for gene length and used TMM correction in edgeR (version 3.36.0) for both pseudobulk and bulk samples. The TISCH2 scRNA-seq data were not further processed.

### Definition and Scoring of Gene Expression Signatures

Gene expression signatures were studied using methods recently published in the context of our RosettaSX platform for expression signature investigation (27). Besides the signatures described in ref. 27, we included two gene expression signatures to describe the CMS4 subtype classifications of the CMScaller and CMSclassifier packages (9, 12). Guinney and colleagues and Eide and colleagues implemented two independent models (CMSclassifier and CMScaller) to classify consensus molecular subtypes (CMS; the CMS4 subtype is associated with EMT; refs. 9, 12). However, there was no signature provided for these subtypes, and consequently, we derived two gene sets that are used for the CMS4 classification. The CMSclassifier package provides a trained random forest model. From the importance table, we selected genes with the highest decrease in mean accuracy for the CMS4 class. Similarly, we extracted the CMS4 template genes from the CMScaller package. In a second step, we identified genes that were significantly upregulated in CMS 4 samples compared with CMS 1–3 samples in TCGA colorectal cancer cohort (one-sided *t* test, log_2_ fold change > 2, adjusted *P* value < 0.05). Overall, this resulted in 139 genes derived for the CMSclassifier model (9) and 33 genes, derived from the CMScaller package (12) which we then used for all analyses. We want to highlight, that these gene sets are used as a proxy for the models, but certainly might not describe the full complexity of the models.

To calculate the signature scores in scRNA-seq data analyses, we used the Seurat addModule function. For signature scores of bulk RNA-seq data, we used the mean TPM of TMM normalized expression, to describe the activity of a signature. For the association statistics, we used Pearson correlation. We used Euclidean distance and complete hierarchical clustering to visualize the heat maps.

### Judging the Coherence and Translatability of Signatures by the Coherence Score

To determine the relevance of the signatures for an expression dataset, we applied the coherence score (CS; refs. 27, 28), and the average Pearson correlation coefficient of all signature gene pairs in a specific dataset. This score indicates a strong negative or positive coherence (CS close to 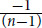 or +1) between all gene expression profiles of a signature or no linear relationship (CS = 0).

In the RosettaSX analysis, we used a CS larger 0.18 to filter for translatable signatures. In previous analyses, we have shown that this threshold can be effectively used to identify signatures with significant coherence of gene pairs (27), even for signatures down to a size of just five genes (*P* < 0.01). For larger signatures with *n*≫5 genes, a CS>0.18 even identifies signatures with stronger significance, and therefore sufficient translatability to a new dataset and interpretability of their per-sample scores.

### Definition of Pseudobulk Sequencing Data

To create pseudobulk RNA-seq data from scRNA-seq data, we applied the SimBu R package (29) and generated pseudobulk expression profiles comprising mixed expression patterns from fibroblast and malignant cells. We sampled a pseudobulk cohort of 20 samples with 20% to 80% fibroblast cells and with the remaining cells being malignant cancer cells. These pseudobulk samples were further analyzed with methods described below. The generation of pseudobulk samples was based on the GSE146771 colorectal cancer dataset.

### Differential Gene Expression Analysis in scRNA-seq Data

For the identification of differential expressed genes in single-cell gene expression data, we used the FindMarkers function of the Seurat package. We filtered for genes that had an average fold change of 2, and a *P* value below 10*e*^–10^ for cells of a type of interest compared with all other available cells.

### Survival Analysis

Outcome data were downloaded from the Pan Cancer Atlas (30). Following the recommendations of the Pan Cancer Atlas study in the disease-free interval (DFI) analyses, we excluded READ and GBM samples from the DFI analyses. Similarly, we included only patients with sufficient stage and tumor purity information, resulting in an exclusion of GBM in the overall survival (OS) analysis. Univariate Cox proportional hazards models were individually fitted to coherent signatures for the individual TCGA indications. Multivariate models were fitted using American Joint Committee on Cancer staging as covariates. *P* values were adjusted for multiple testing using the Holm method. We evaluated for proportional hazards using the Schoenfeld residuals.

### Data Availability

TCGA gene expression RNA-seq data (TCGA RRID: SCR_003193) were downloaded from the Xena database (31). CPE tumor purity scores were assessed using TCGAbiolinks (32) and ABSOLUTE tumor purity from (33). Cell type and cell state abundances and annotations were accessed from ref. 34 and https://ecotyper.stanford.edu/carcinoma/. Cancer cell line data were downloaded from DepMap (ref. 35; release 20q4, RRID: SCR_017655). For cell line annotations, we used integrated Cellosaurus cell line annotations (36).

We downloaded scRNA-seq data (EMTAB8107, GSE148673, GSE161529, EMTAB8107, GSE146771, GSE166555, GSE141383, and GSE103322) from the TISCH2 database (37).

## Results

**FIGURE 1:**
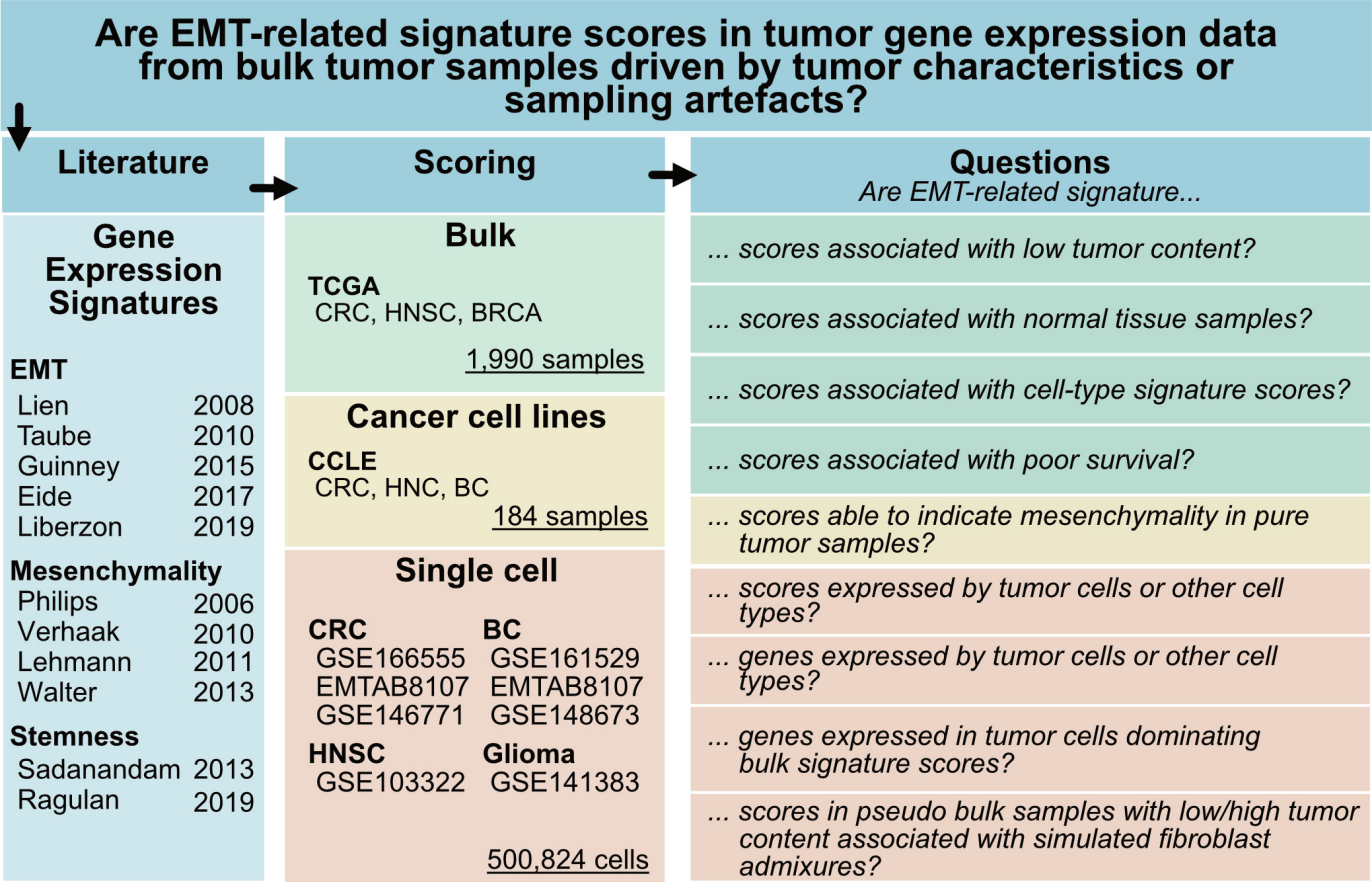
Workflow for delineating the influence of non-tumor cells on EMT-related signature scores. We identified 11 frequently applied gene expression signatures from literature that were proposed as markers for stemness or mesenchymality character of tumors. We investigated the signatures in gene expression data of clinical samples, cancer cell lines, and single cells to address questions related to their utility for clinical cancer profiling. CCLE: Cancer Cell Line Encyclopedia, EMT: epithelial-to-mesenchymal transition, TCGA: the cancer genome atlas.

Previous studies have shown that the expression profiles of individual EMT-related signatures across clinical tumors are often similar but could be strongly influenced by noncancerous cells in the TME or simply by tumor sample composition (tumor macroenvironment, normal stromal tissue surrounding a tumor). We used the workflow shown in Fig. 1 to analyze 11 different published EMT, mesenchymal, and stemness gene expression signatures (hereafter referred to as EMT signatures) in bulk tumors, cell lines, and single-cell gene expression data to identify the factors that primarily drive their scores (2–12).

First, we assessed the association of 11 EMT signature profiles with cancer cell content across samples and analyzed them in the context of a literature-derived collection of gene expression signatures in our RosettaSX system (27). We focused on the analysis of the colorectal cancer, BRCA, and HNSC bulk RNA-seq data. Our signature collection enabled us to explore the associations between EMT signatures and other signature profiles for a variety of cancer-relevant processes. Next, using normal tissue adjacent to the tumor (NAT), cancer cell line bulk RNA-seq, scRNA-seq, and simulated pseudobulk data, we investigated the contribution of noncancerous cells from the macroenvironment or microenvironment of tumors to EMT-related signature scores.

### EMT-related Signature Scores are High in Samples with Low Cancer Cell Content

**FIGURE 2:**
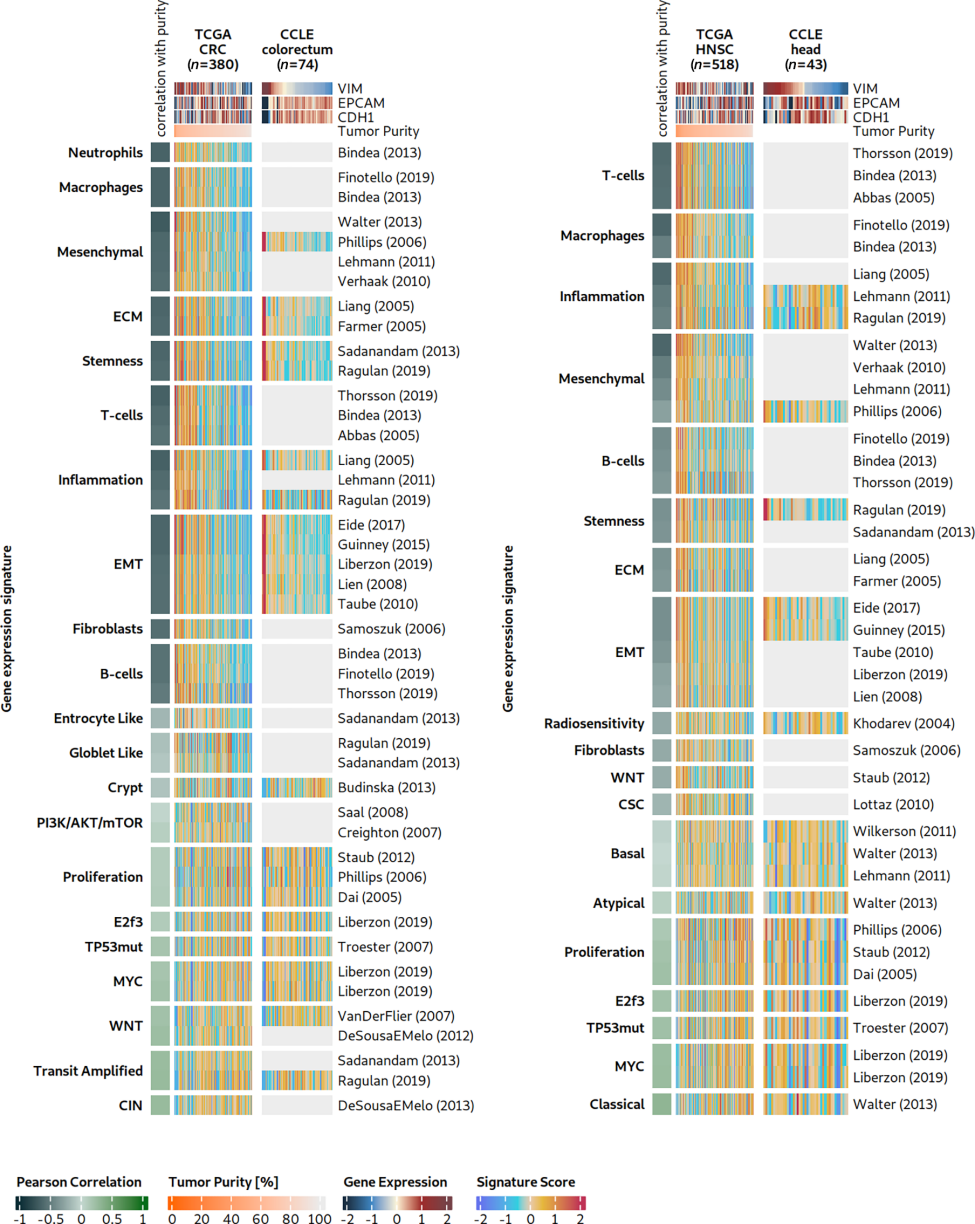
EMT-related signatures and their associations with cancer pathways or processes. We studied 11 mesenchymal, EMT, and stemness signatures and their relation to other biological processes in TCGA cancer patients and CCLE cancer cell lines. Left heat maps: Gene expression signature scores of coherent signatures and their correlation with sample purity (CPE) in TCGA colorectal cancer (CRC) samples. The heat maps have been supplemented with single gene marker expression. The column annotation lists important markers for mesenchymal (*VIM*) and epithelial (*EPCAM*, *CDH1*) properties. Samples from left to right have decreasing levels of *VIM* expression, and signatures groups have an increasing correlation with tumor purity from bottom to top. Light gray areas indicate signatures that did not satisfy the coherence scoring threshold. Right heat maps: An analogous analysis as in colorectal cancer to the left, here for TCGA HNSC samples.

Bulk RNA-seq tumor samples can comprise high fractions of tumor-adjacent normal tissue cells, especially stromal and immune cells, if no macrodissection or microdissection is performed by pathologists during sample preparation. Stromal or immune cells of tumor-adjacent tissues can potentially influence gene expression signals more strongly than the characteristics of cancer cells themselves. Thus, we compared TCGA provided cancer cell content with signature scores in TCGA colorectal cancer population (Fig. 2, left; Supplementary Fig. S1A and S1B). EMT and immune cell type signature scores were strongly anticorrelated with cancer cell content [rMED(EMT) = −0.62 and rMED(IMM) = −0.62], whereas the other signatures were weakly positively correlated with cancer cell content (rMED = 0.11). A comparison of the 11 EMT signatures with the 22 cancer process signatures revealed that EMT signatures had a stronger negative association with cancer cell content than any other process (excluding cell type signatures, one-sided Wilcoxon test, *P* < 0.0001, effect size = 0.7). In addition, there was a strong correlation between the signature scores of EMT-related signatures and the stromal signature for BRCA (R between 0.83 and 0.98; signature from ref. 38). These findings suggest that the presence of non-tumor cells from the stroma significantly drives, if not dominates, EMT-related signature scores. Analyses of other cancer types [HNSC, BRCA, GBM, and pancreatic adenocarcinoma (PAAD)] represented in TCGA yielded comparable results (Fig. 2, right; Supplementary Fig. S2–S4).

Summarizing our results on the bulk gene expression data of patients with cancer, we found that the collection of 11 EMT- or stemness-related signatures (i) each had a stable expression footprint (high CS) in bulk gene expression data, (ii) shared very similar signature profiles in clinical tumor specimens despite differences in the procedures for their discovery and their gene sets, (iii) were significantly associated with low cancer cell content, and (iv) all correlated strongly with a well-known signature to track stromal content in BRCA. These findings motivated us to further investigate the 11 EMT signatures in pure tumor cells (cancer cell lines) and single-cell gene expression datasets to find out how much of the mesenchymal expression signal comes from cancer cells versus stroma cells.

### Diverse EMT, Mesenchymal, or Stemness Signatures Generate Highly Congruent Signature Profiles in Patients with Primary Cancer

To assess the signature landscape of colorectal cancer, we analyzed 380 TCGA colorectal cancer tumors (TCGA COAD and READ) using RosettaSX. We applied the CS (27, 28) to determine the within-signature congruence of gene expression profiles. The CS indicates whether genes in a gene expression module are coordinately activated across patients. This indicates whether a given signature is coherently active in some samples while being inactive in others. Only signatures with high CSs should be used to infer the activity of their underlying biological phenomena in a single sample of the dataset to be explored. A total of 121 of the 285 RosettaSX signatures were coherently expressed (CS > 0.18). From these, we selected signatures relevant to known cancer-related processes (e.g., proliferation, and colorectal cancer subtypes), cellular composition of the tumor sample, and EMT- or stemness-related signatures. Furthermore, we filtered out signatures with a Jaccard index larger than 0.25 to other signatures to remove overly redundant biological processes. Thereby, we derived a set of 44 signatures that comprise the 11 published EMT signatures and further 33 signatures that cover other biological phenomena, such as the expression of extracellular matrix (ECM) or proliferation genes, or the presence of specific cell types, such as T, B cells, or fibroblasts (Fig. 2, left side).

The CS analysis revealed that the 11 EMT-related signatures had a median (MED) CS of 0.41 [interquartile range (IQR) = 0.26], whereas the remaining 33 signatures reached a MED CS of 0.32 (IQR = 0.22). Thus, genes of EMT-related signatures are among the signatures with the strongest gene coexpression, indicating a coordinately regulated expression pattern that is presumably guided by a strong underlying biological phenomenon involving the signatures’ genes. We found that EMT-related signature scores were more strongly correlated with common mesenchymal markers than with epithelial markers (one-sided Wilcoxon test, *P* < 0.0001, effect size = 0.85), confirming a predominantly mesenchymal characteristic of mesenchymal-related signatures as well as stemness and EMT signatures. In the colorectal cancer bulk RNA-seq data, EMT-related signature profiles were strongly associated with signatures describing the presence of different noncancerous cell types [macrophages (39), fibroblasts (40), or T cells (41)] and genes encoding the ECM (refs. 38, 42; Fig. 2, left; Supplementary Fig. S5). These associations might indicate that cells in the tumor environment (microenvironment or tumor-adjacent normal macroenvironment) strongly contribute to EMT-related signature scores. We observed a low or anticorrelation between gene expression signatures and phenomena such as MYC activation (11), proliferation (28, 43), and colorectal cancer subtypes or cell-of-origin (e.g., goblet-like, crypt, or CIN) gene expression signatures (refs. 2, 3, 44; Supplementary Fig. S5).

In TCGA HNSC gene expression data, 40 signatures met the previously described selection criteria (Fig. 2, right). Their signature scores recapitulated our findings regarding colorectal cancer. EMT-related signatures were more strongly correlated with non–cancer cell type signatures (e.g., T-cell and B-cell) than with cancer-associated processes (e.g., proliferation, and MYC activation). Similarly, EMT-related signatures were more strongly correlated with mesenchymal markers than with epithelial markers (one-sided Wilcoxon test, *P* < 0.0001, effect size 0.85). Analogous analyses of TCGA BRCA, GBM, and PAAD data yielded comparable results (Supplementary Fig. S2–S4).

In summary, these results suggest that for samples with high signature scores either cancerous cells have lost their epithelial expression phenotype or that noncancerous cells in the tumor macroenvironment or microenvironment contribute strongly and sometimes even dominantly to EMT-related signature scores. Therefore, we decided to further investigate whether the signals of EMT signatures are associated with tumor sample impurities, that is, low cancer cell content of some samples.

### EMT, Mesenchymal, and Stemness Signatures are Less Congruent in TME-naïve Cancer Cell Line Models than in Bulk Samples

To investigate whether TME-naïve pure cancer models (lacking immune or stromal cells) show signs of differential EMT states or mesenchymality/stemness, we analyzed our set of EMT-related gene expression signatures in colorectal cancer cell line data (Fig. 2 left, right heat map). Of the 44 signatures we previously selected for colorectal cancer TCGA samples, only 22 passed our quality control procedure based on the CS. As expected, many cell type gene sets (e.g., T cells, B cells, and macrophages) have low CSs, and consequently, interpretations of signals of these signatures in the cell line data cannot be regarded as meaningful.

However, EMT-related signatures yielded acceptable CSs. Our analysis revealed a small group of cell lines with extraordinarily high signature scores for the 11 EMT-related signatures and signatures related to the ECM or fibroblasts (Fig. 2B; Supplementary Fig. S1C). These cell lines exhibited low epithelial marker expression (*EPCAM*) and high mesenchymal marker expression (*CDH1*). Their results warrant follow-up to investigate how these EMT-high cell lines were established and what their cellular origin could be. A review of their annotations revealed that three cell lines originated from mesenchymal cells but not carcinoma cells (HS255T, HS675T, and HS698T; from fibroblasts or from a gastric tumor, presumably a gastrointestinal stromal tumor). Removal of these cell lines and restriction of the analysis to carcinoma cell lines drastically worsened the CSs, with an average CS decrease of 0.11 in the EMT signatures (Supplementary Fig. S6) as now high expressing cell lines were absent and could not drive the correlation. This indicated that the higher CSs in the data for all 44 cell lines were primarily driven by cell lines that originated from mesenchymal cancers, not typical colorectal carcinomas. This not only exemplifies the utility of the CS approach, but also further questions whether the investigated EMT signatures can capture expression signals of mesenchymality that are cancer cell–intrinsic.

### The Tumor Macroenvironment can Influence EMT-related Signature Scores

**FIGURE 3:**
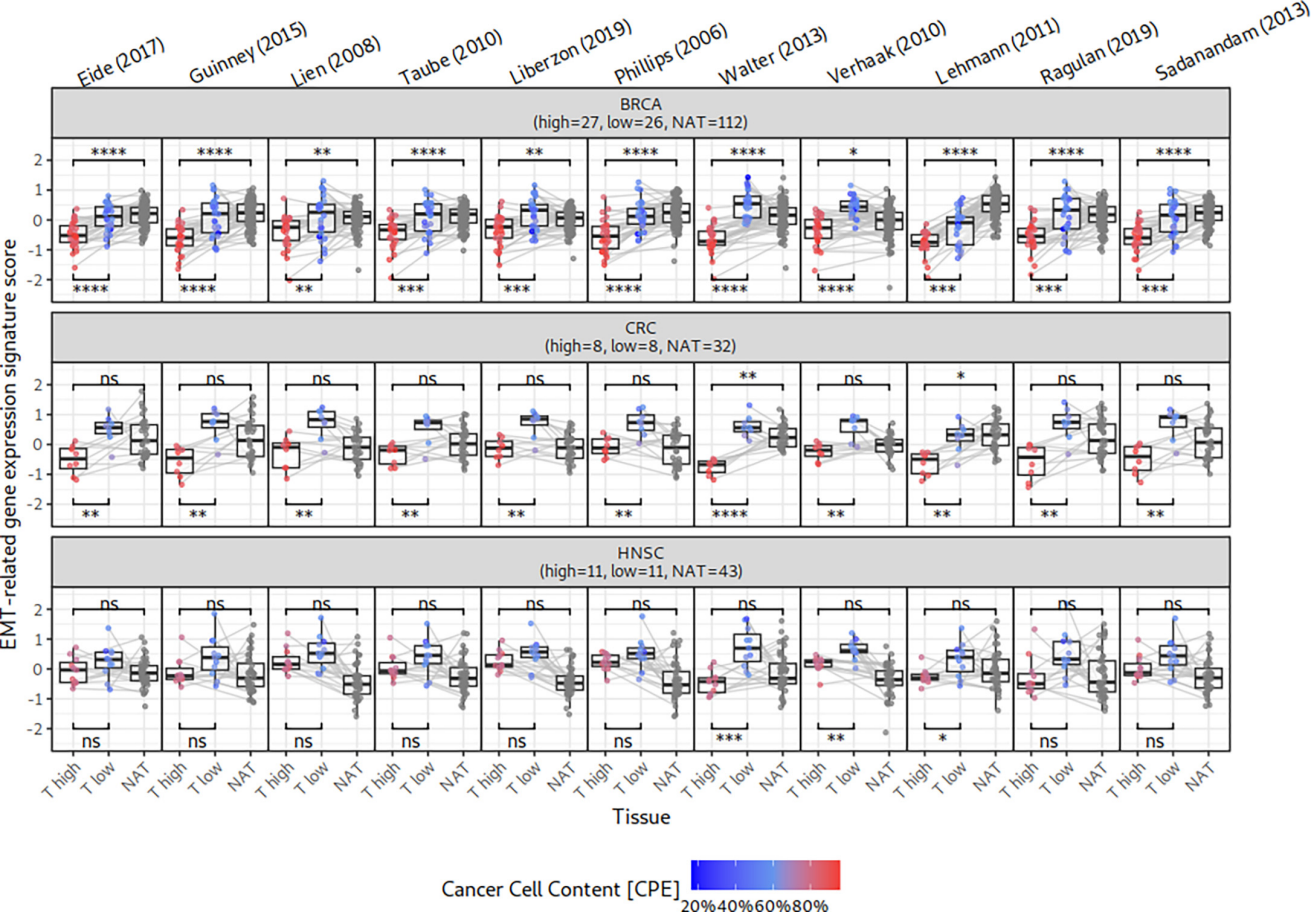
Comparison of EMT-related gene expression signature scores in tumor and normal tissue that is adjacent to the tumor tissue. Tumor samples are separated into low, medium, and high cancer cell content (CPE) based on the 25th (low) and 75th (high) percentiles of TCGA cancer cell content estimates. Lines indicate the paired tumor (T) and normal adjacent to tumor (NAT) samples. Colors indicate the cancer cell content in tumor samples with low (blue), or high (red) cell content or gray for missing data. Scores in NAT and high cancer cell content samples were compared using one-sided paired *t* tests for the “T high” and NAT comparison and a one-sided *t* test for the “T high” versus “T low” comparisons. *P* values were adjusted using Bonferroni correction (0 = ****, <1e-4 = ***, <0.001 = **, <0.05 = *, ns).

To further dissect the influence of cancer cell–extrinsic signals, we compared signature scores in high tumor content samples (upper quartile), low tumor content samples (lower quartile), and NAT samples (Fig. 3). We note here, as it is important for interpreting the following results, that the high tumor content group has various levels of tumor content across TCGA cohorts. We observed a maximal cancer cell content of 96.01%, 95.69% in BRCA and colorectal cancer, and a lower maximal tumor content of 85.95% in HNSC.

When comparing the high and low cancer cell content groups, we observed higher EMT/mesenchymal signature scores in the low group (one-sided *t* test; Fig. 3, bottom group comparisons). For most mesenchymal signatures (4, 5, 7), the scores were significantly higher in the low compared with the high cancer cell content group across all analyzed indications. We noted a statistically significant increase in scores of all signatures with decreasing tumor content in BRCA and colorectal cancer. For HNSC (the indication with the overall lowest cancer cell content across samples) for all signature scores, there was at least a nonsignificant trend toward higher scores in the low cancer cell content groups. For BRCA we also found that the scores in NAT are often higher than both low/high tumor content groups, and often more like the low tumor content group than to the high tumor content group. For colorectal cancer and HNSC, hardly any significant differences were found for the NAT group compared with others, possibly due to lower sample number, or less stromal content in NAT samples of colorectal cancer and HNSC compared with BRCA. In summary, these differences suggest a major contribution of the TME or macroenvironment to scores of EMT-related signatures.

To further differentiate the influence of individual cell types in the microenvironment and macroenvironment, we analyzed the associations between signature scores and precomputed cell type proportions in paired tumor and NAT samples across TCGA cohorts (as accessed from results of expression-based cell type deconvolution of original authors in ref. 34). We correlated cell type abundances across the different TCGA cohorts with gene expression signature scores, to identify associations with specific cell types (Supplementary Fig. S7–S10). We observed a high correlation between the signature scores and cell types that were described to be specifically enriched in NAT tissues across all indications (CAF2, endothelia cells, epithelial cells; Supplementary Fig. S7–S10, Normal column). In tumor samples, proangiogenic epithelial, and tumor-associated fibroblasts (CAF1) and endothelial cells were most strongly correlated with the signatures. However, the second most associated cell types were the same cell types we observed in the NAT samples (Supplementary Fig. S7–S10, Tumor column). This indicated a considerable contribution of cell types that are enriched in normal tissue to EMT-related signature scores in tumor samples. Even though the analyses of CAFs agree with our previous findings, we note that the associations of proangiogenic cell type abundances must be interpreted with caution. As described by the original publication, *COL1A1* is strongly associated with a proangiogenic cell type state. However, previous studies in patients with ovarian, gastric, pancreas, lung, and renal cancer or cell lines (45–49), indicated a profound expression of *COL1A1* by CAFs or myofibroblasts. Thus, it is likely that proangiogenic cell state abundances are partially driven by certain types of fibroblast lineages. This would also be in alignment with CAFs of the tumor environment being responsible for the association of EMT-related signature scores and proangiogenic cell abundances.

Overall, these results provide further evidence that across indications, EMT-related signatures are driven by cells of the TME or macroenvironment. For BRCA, there is additional evidence that the macroenvironment (with NAT as a proxy) can be a source of high EMT-related signature signals.

### Single-cell Gene Expression: Cell Type Composition Strongly Affects Scores of EMT-related Signatures

To further investigate the origin of elevated EMT-related signature scores in bulk tumor specimens, we analyzed their signature scores and individual signature gene expression levels in eight scRNA-seq datasets derived from colorectal cancer, BRCA, glioblastoma, and HNSC samples (see Materials and Methods).

**FIGURE 4:**
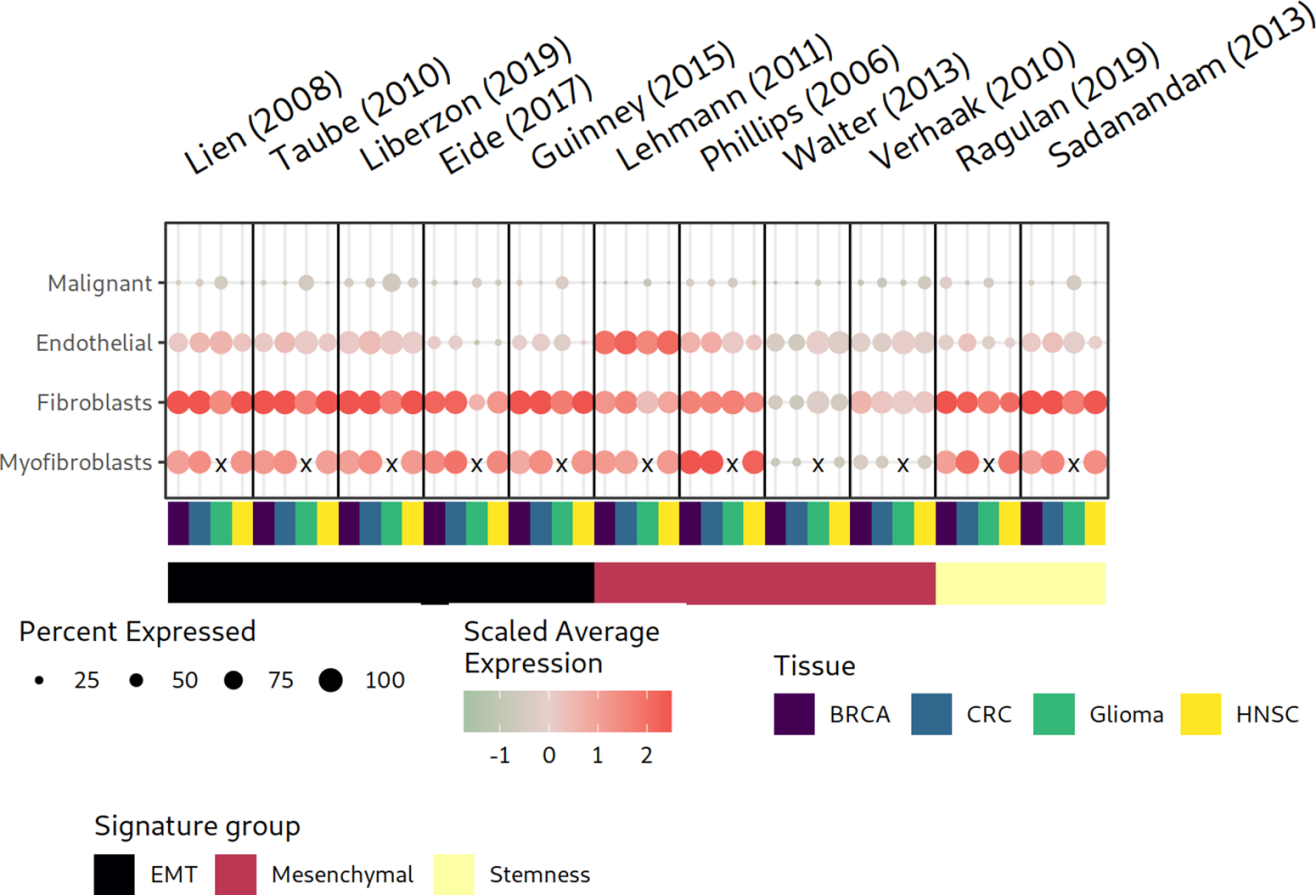
Comparison of EMT-related signature scores in eight scRNA-seq datasets. The dot plot shows the module scores of EMT-related signatures and the number of cells expressing them in the different cell types. Each panel displays dot plots to quantify the expression of individual signatures in four different tissues (malignant, endothelial, myofibroblast, and fibroblast) derived from scRNA-seq datasets for BRCA, colorectal cancer (CRC), glioma, and HNSC. X, Unavailable cell type.

First, we examined the expression of EMT-related signature scores within the individual cell types (Fig. 4). With high consistency across the four analyzed cancer indications, we found that all EMT-related signatures displayed the highest average expression in cell populations of the TME, fibroblasts, myofibroblasts, or endothelial cells, but not in malignant cells. These high expression levels in fibroblasts underpin the overall dependency of the EMT signatures on stromal cell signals but not on cancer cells. Overall, only a fraction of the cancer cells expressed EMT-related signatures at low levels. We cannot assess whether these single cells are from the TMEs or macroenvironments, but in combination with previous results this is convincing evidence that scores of all 11 signatures are heavily influenced or even dominantly driven by fibroblasts or other noncancerous cells.

### High EMT-related Signature Scores are Associated with Fibroblast-enriched Pseudobulk Samples

**FIGURE 5:**
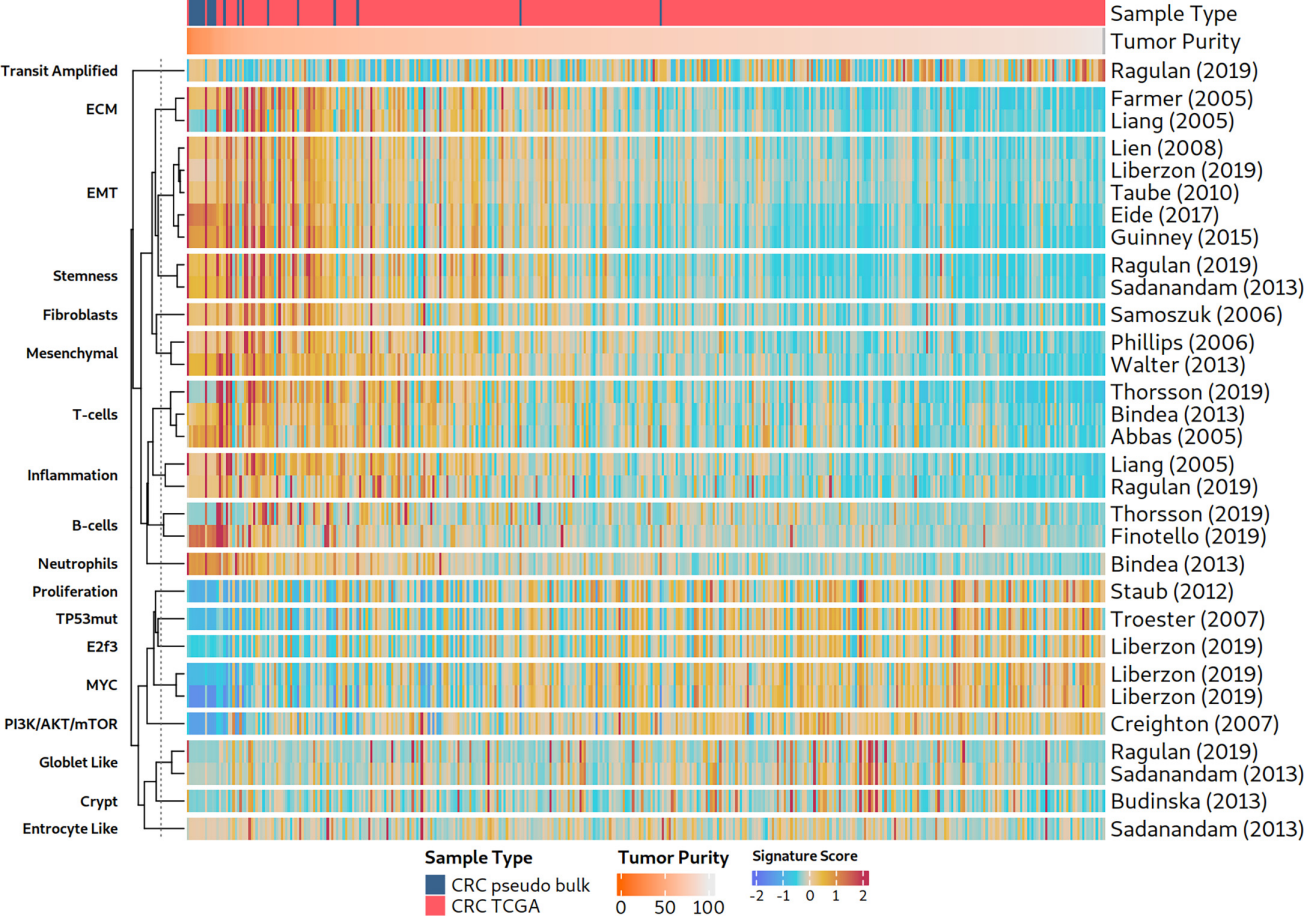
RosettaSX analysis of an integrated dataset of TCGA colorectal cancer (CRC) and fibroblast-enriched pseudobulk samples. Top annotation indicates the tumor purity (CPE) for TCGA samples, and the fraction of fibroblast cell RNA used for pseudobulk generation (see Materials and Methods). The tumor samples (columns) are sorted by their tumor purity from low (left) to high (right). Note the tight clustering of all 11 EMT-related signatures and their correlation with tumor purity.

If tissue biopsies are used in combination with bulk sequencing experiments, we hypothesized that minor sampling artifacts can strongly influence signature scores depending on the fraction of sampled fibroblasts. To further study the dependency of EMT-related signature scores on the fraction of fibroblast cells, we generated 20 pseudobulk RNA-seq samples based on colorectal cancer scRNA-seq data [EMTAB8107 (37)]. Each pseudobulk sample was composed of RNA counts from different ratios of tumor to fibroblast cells, ranging from 20% to 80% of cancer cell content (see Materials and Methods). We investigated the similarities of RosettaSX pathway and process signatures in an integrated analysis of 20 pseudobulk and 380 TCGA colorectal cancer bulk samples using an integrated RosettaSX analysis (see Materials and Methods; Fig. 5). Pseudobulk samples with enriched fibroblast content coclustered with samples with low cancer cell content they had elevated EMT-related, stroma, or TME cell type signature scores, indicating that fibroblasts might significantly drive the signature scores of the analyzed bulk RNA-seq samples and drive coclustering. In contrast, bulk samples with high cancer cell content had elevated scores for proliferation or colorectal cancer subtype signatures [e.g., transit amplified (3), and crypt (50)]. Our results showed that high EMT-related signature scores in low-purity TCGA samples can result from abundant fibroblast cells in the low cancer cell content samples and do not necessarily originate from the mesenchymality of the cancer cells.

### Fibroblasts Dominate the Expression for Most Genes in EMT-related Signatures

Furthermore, we investigated the cell-type specificity of the individual signature genes (Table 1). Overall, the results of our investigations of cell type–specific expression of the signature genes were congruent across the analyzed colorectal cancer, breast, HNSC, and glioma scRNA-seq datasets. Most of the cell types that expressed individual signature genes were fibroblasts, but not tumor cells (Supplementary Fig. S11). Differential gene expression analysis of all available signature genes within the eight scRNA-seq datasets confirmed the low contribution of tumor cells to the signature scores (see Table 1). Overall, only a small number of genes showed significantly higher expression in cancer cells than in fibroblasts. Only four signatures for mesenchymal and EMT characteristics indicated a higher expression of a few genes (a maximum of 3.7% of individual signature genes) in cancer than in other cell types in at least one dataset (4, 5, 8, 11). These patterns highlight that fibroblasts were the primary contributors to the high overall signature scores for our set of EMT-related signatures. Thus, these EMT-related signatures often predominantly describe mesenchymal characteristics of the tumor macroenvironments or microenvironments rather than the stemness or mesenchymal character of cancer cells when bulk tumor tissue is profiled, especially when the cancer cell content is low, and the fraction of adjacent normal tissue is high.

**TABLE 1:** Differentially expressed EMT-related signature genes between fibroblasts and cancer cells in summarized across three (BRCA), three (CRC), one (glioma), and one (HNSC) scRNA-seq datasets

Gene set	BRCA^a^	CRC^a^	Glioma^a^	HNSC^a^
EMT				
Eide (2017)	2.1 (0.0)	2.6 (0.0)	0.0 (0.0)	5.6 (0.0)
Guinney (2015)	6.7 (0.0)	8.3 (0.0)	0.0 (0.0)	10.5 (0.0)
Liberzon (2019)	25.5 (0.2)	29.0 (0.0)	13.2 (0.5)	18.4 (1.0)
Lien (2008)	38.3 (0.0)	45.7 (0.0)	14.8 (0.0)	33.3 (0.0)
Taube (2010)	25.2 (0.4)	26.6 (0.0)	17.3 (0.0)	18.2 (1.1)
Mesenchymal				
Lehmann (2011)	3.4 (0.0)	5.7 (0.0)	2.9 (0.0)	3.5 (0.0)
Phillips (2006)	56.4 (0.0)	64.1 (0.0)	38.5 (0.0)	38.5 (0.0)
Verhaak (2010)	3.7 (0.0)	4.1 (0.0)	2.6 (0.0)	2.6 (0.7)
Walter (2013)	5.6 (0.3)	6.3 (0.0)	2.8 (0.0)	5.2 (0.4)
Stemness				
Ragulan (2019)	44.4 (3.7)	53.7 (0.0)	25.0 (0.0)	44.4 (0.0)
Sadanandam (2013)	27.4 (0.5)	35.7 (0.0)	8.2 (0.5)	25.9 (0.0)

^a^DEG fibroblast % (DEG malignant cells %).

### Cancer Cell–specific Gene Expression does not Dominate Bulk Sequencing Signature Scores of EMT-related Signatures

**FIGURE 6:**
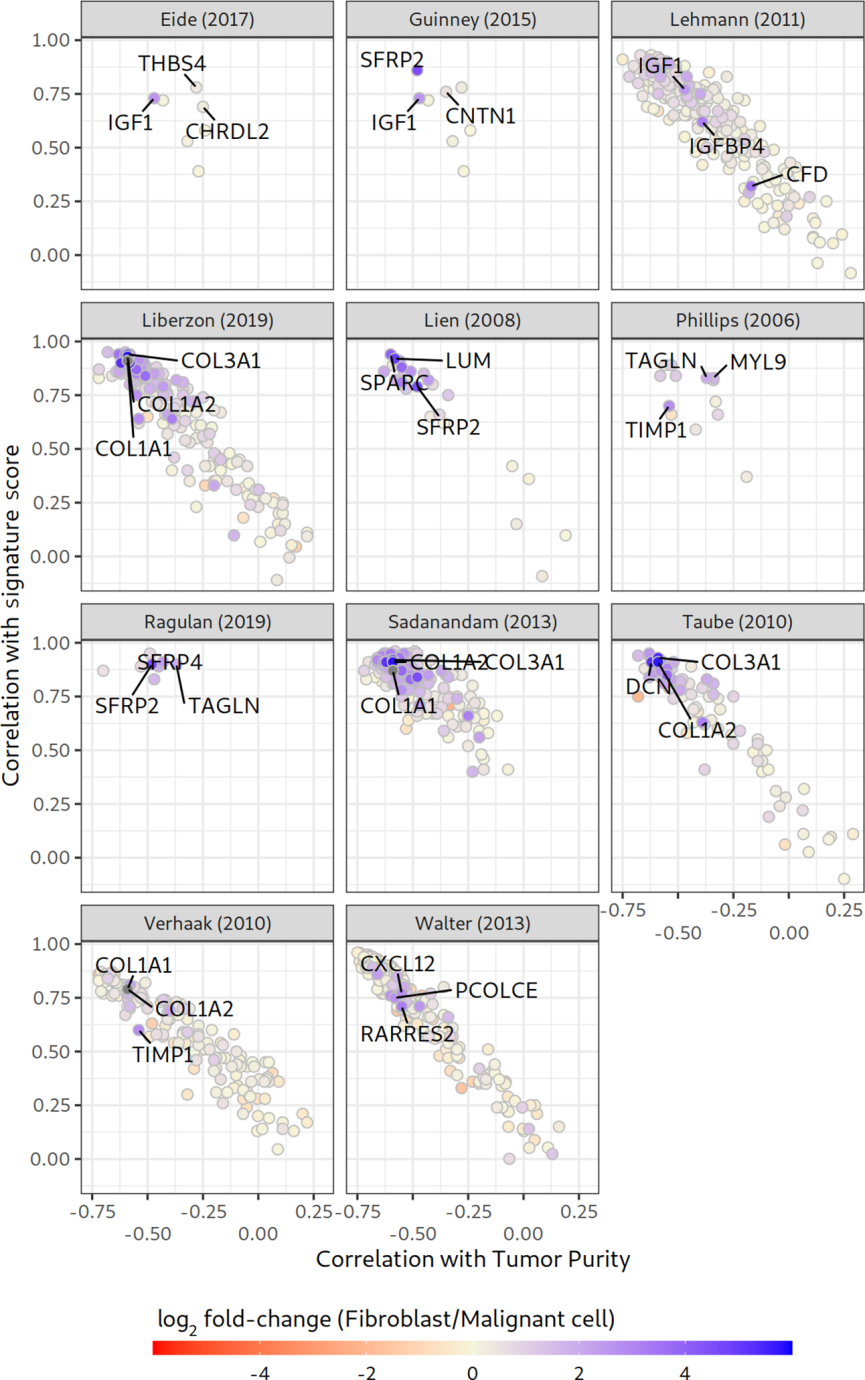
Influence and association of signature gene expression profiles on signature scores in colorectal cancer (CRC) bulk RNA-seq samples. Each dot in a panel represents a signature gene of the respective signature. A gene at the bottom weakly influences the overall signature score. A gene at the top strongly influences the overall signature score. Genes on the left are stronger correlated with low cancer cell content. Blue coloring of data points indicates a higher expression in fibroblasts than in malignant cells in our scRNA-seq analyses. Labeled genes are the top three genes with the highest expression in fibroblasts.

Our single-cell analysis showed a high expression of most EMT-related signature genes in the TME cells. Thus, it is likely that the signature score profiles in bulk sequencing data are primarily driven by TME cells. To translate the findings of our single-cell analysis to bulk sequencing experiments, for each signature gene, we compared three variables: (i) expression fold changes between tumor and fibroblast cells of scRNA-seq data, (ii) the correlation of the gene expression profile with tumor purity (bulk RNA-seq data), and (iii) the correlation of the gene expression profile with the respective signature scores in patients with TCGA colorectal cancer (Fig. 6).

Overall, genes strongly associated with the respective signature scores (in bulk sequencing data) were expressed at higher levels in fibroblasts than in tumor cells in the scRNA-seq analysis. In addition, at the bulk sequencing level, genes with high association with the respective signature score and high fibroblast expression were associated with low tumor purity. These patterns indicate that genes associated with low tumor purity significantly contribute to EMT-related signature scores, and that fibroblasts express many of these genes at a higher level than tumor cells (Fig. 6). The genes that were highly expressed in fibro-blasts were *VIM*, *COL1A1*, *COL1A2*, and *LUM*, which have been previously associated with fibroblasts (51, 52).

### Our Analyses do not Confirm a Prognostic Value of EMT-related Gene Expression Signature Scores

Some EMT-related gene expression signatures have frequently been postulated to be associated with poor survival in different indications (2, 19, 20). However, other studies could not verify a universal correlation across cancer indications (53, 54). In addition, often these results arise from arbitrary selected cutoffs for the classification of high or low expressing samples (55), combined and pruned published gene expression signatures for the most prognostic genes (19), or did not provide sufficient information of data sources (55). Therefore, the analysis might result in false or overoptimistic conclusions (56, 57), describe other phenomena or might rely on insufficient data (34).

**FIGURE 7:**
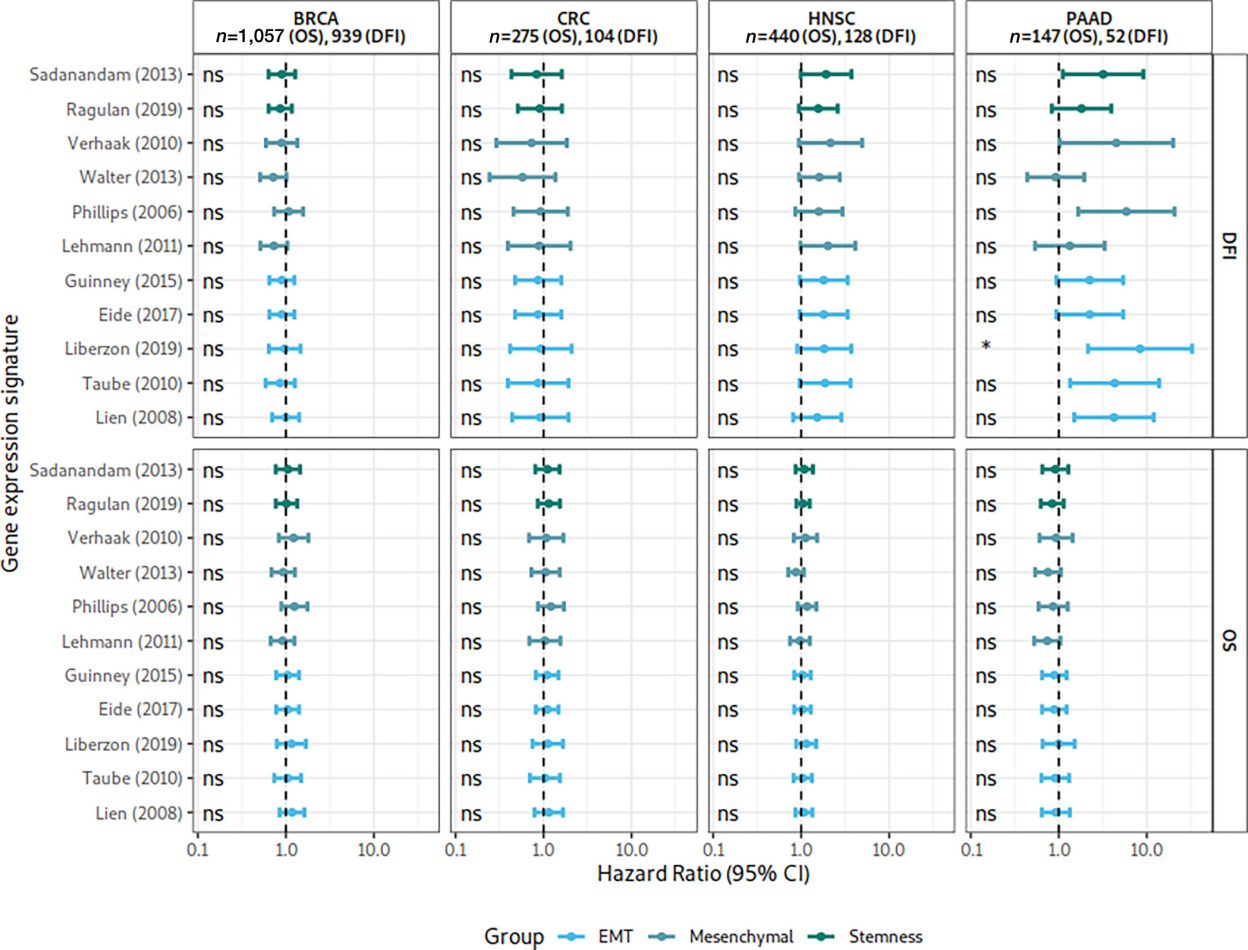
Univariate Cox proportional hazards analyses for EMT-related signature scores and TCGA outcomes. *P* values were adjusted for multiple testing within each panel using the Holm adjustment method. Top: Association with DFI, excluding GBM and READ due to short follow-up or small number of events. Bottom: Association with OS, excluding GBM due to missing clinical readouts. ns, not significant; **, *P* < 0.01; *, *P* < 0.05; ns, *P* > 0.05.

Thus, we re-evaluated the prognostic value of the analyzed EMT-related signatures. We compared the association of signature scores with DFI and OS, in patients with colorectal cancer, BRCA, HNSC, and PAAD TCGA (Fig. 7). In a univariate Cox proportional hazards model, none of the signature scores were associated with the survival outcomes. There is the exception of one EMT signature from Liberzon and colleagues (11) in PAAD that yielded a significant association with DFI, but not OS: There was a trend for worse DFI in HNSC and PAAD for several high signature scores. Most associations were not significant after multiple testing correction. As a second step, we added tumor stage and cancer cell content as confounding factors in a multivariate Cox proportional hazards model (Supplementary Fig. S12). For colorectal cancer, BRCA, HNSC the signature remained not to be associated with outcome measured by OS or DFI. Interestingly, for PAAD, all EMT, one mesenchymal and one stemness signature had a higher HR and *P* value estimates were significant after multiple testing correction for DFI. This is evidence for EMT-related signature scores being relevant, solely in PAAD for DFI, but not for OS. We note that this analysis does not yield information whether the presence of intermixed cancer and fibroblasts or the presence of a cancer cell–intrinsic EMT character is causative for association with DFI.

## Discussion

This study examined the impact of the cell type composition of a tumor sample on 11 established gene expression signatures that are proposed to describe stemness, mesenchymal, and EMT cancer subtypes (2–12). It is noteworthy that not all the investigated EMT-related signatures have been proposed to serve as readouts of tumor mesenchymality in clinical samples. Previous studies revealed that cell type composition severely affects published transcriptional cancer subtyping procedures with EMT-related characteristics across multiple cancer indications (14, 19, 20, 22, 24). However, a global examination of the affected signatures and cancer indications was lacking.

We found that across cancer indications, the discussed signatures reveal a strong footprint of epithelial versus mesenchymal characteristics in clinical samples, as indicated by high CSs (Fig. 2; Supplementary Fig. S2–S4). These high CSs were observed in the original cancer indications, in which the signatures were developed, and in other indications. The key question remains as to whether this signal results from differences in tumor characteristics (cancer cell–intrinsic) or the number of fibroblasts sampled into a tumor specimen (from the TME or from neighboring normal tissue). The cancer cell content of a clinical tumor sample is strongly associated with high EMT-related signature scores (Fig. 2; Supplementary Fig. S2–S4, left). It became evident that biopsies with cancer cell content are less frequently associated with EMT-related phenomena across different cancer tissues than macroenvironment or tumor samples with low cancer cell content. This finding was corroborated by our analysis of TME-naïve cancer cell lines (Fig. 2; Supplementary S2–S4, right). The EMT/stemness expression footprint was reduced across cancer indications when stromal or fibroblast cell lines were excluded from the analysis (Supplementary Fig. S6). Although previous studies highlighted the dependence of signature scores on the cancer cell content of colorectal cancer and BRCA samples (15, 20, 58), a comprehensive analysis of cancer indications and established EMT-related signatures was lacking. Our findings indicate that, as a group, these signatures indeed have significant and steady expression footprints; however, they suggest that sampling of fibroblasts from the tumor macroenvironment and microenvironment is the reason for high scores and that cancer cell–intrinsic mesenchymality can hardly be assessed reliably with these signatures in clinical samples (Fig. 3; Supplementary Fig. S7–S10). In other words, the fibroblast-rich cell type composition of a sample with low cancer cell content might disguise the true cancer phenotype of a clinical tumor sample, eventually affecting the prevalence and characteristics of the other cancer subtypes. Although some articles acknowledged this issue for a subset of the analyzed signatures (14), there is no general recognition of this problem. We show here that assessment of a mesenchymal state in bulk tumor tissue is error-prone for the most widely known gene expression signatures. We hypothesize here that this phenomenon could affect expression-based subtyping schemes in many other indications, in which the existence of mesenchymal/stemness phenotypes has been postulated (59, 60).

Consistent with the results in BRCA (20), colorectal cancer (19–22), HNSC (14), and lung and pancreatic cancer (24), scRNA-seq data indicated that none of the analyzed EMT-related signature scores, and only a minority of signature genes were elevated in tumor cells but in fibroblasts (Fig. 4; Table 1; Supplementary Fig. S11). Our integrated RosettaSX analysis of bulk RNA-seq and simulated pseudobulk samples highlighted that low tumor and fibroblast-enriched pseudobulk samples had elevated EMT-related and ECM/fibroblast signature scores (Fig. 5). In bulk RNA-seq samples, fibroblasts may contribute primarily to the elevated signature scores (Supplementary Fig. S7–S10). Recent studies described leader cells with a hybrid EMT state that can initiate the migration of cancer cell clusters to metastatic sites (61, 62). This study does not invalidate such a phenomenon, as the number of patients and sampling procedures limited our scRNA-seq analyses. However, it may fail to recognize individual cancer cells with EMT-related phenotypes because a few cancer-specific signature genes cannot guide the signature profile in a complex cell mixture of low cancer cell content bulk RNA-seq samples. Our integrated gene expression signature analysis showed that samples with low cancer cell content closely align with profiles of fibroblast-enriched pseudobulk samples, both displaying higher EMT-related signature scores than in any sample with high cancer cell content (Fig. 5). In samples with low cancer cell content, EMT-related signature scores are driven by the fraction of tumor-surrounding cells, primarily fibroblasts. Thus, accurate control of the cancer cell content is required to determine cancer-specific EMT-related phenomena using gene expression signatures.

Our results indicate that none of the analyzed signatures primarily describes cancer cell–intrinsic characteristics. The deconvolution of NAT and tumor samples highlights a possibly strong contribution of cell types that are especially enriched in the tumor macroenvironment or microenvironment (endothelial cells, CAF2 and CAF1, tip cells, proangiogenic epithelial cells, Supplementary Fig. S7–S10). We note that association of fibroblasts might be even more significant, as the deconvolution might be distorted by inaccuracies of the deconvolution approach. Even though the deconvolution analysis indicated a broad contribution of many TME cells, our single-cell analysis could not recapitulate these associations. Similarly, cell type states that were strongly associated with the signature scores (epithelial proangiogenic), have been described to be associated with the expression of *COL1A1*, which was shown to be strongly expressed in fibroblasts, as indicated by our single-cell analysis (Fig. 6) and others (45–49). These results question the availability of a signature that can describe EMT-related phenomena of cancer cells in bulk gene expression data. Such a signature, however, would be required for a sophisticated deconvolution method that is able to separate cancer cell–intrinsic mesenchymality signals from signals originating from fibroblasts of the macroenvironment that are sampled at varying frequencies.

In colorectal cancer, research groups reinterpreted the influence of fibroblast content on EMT-related subtypes as a sign of fibrosis (9, 19, 20), rather than an artifact of the sampling procedure. The main argument for fibrotic activity being a tumor trait, and thus an EMT-related subtype, is an association between patient survival and the net signature assignment in survival analyses. As Venet and colleagues researchers already noted similarly, the existence of a survival association is often a bystander effect and can hardly be interpreted as evidence for the importance of a biological phenomenon in the cancer context (63). Apart from that, we could not verify an association between patient survival and EMT-related signature scores in untreated patients by using TCGA for colorectal cancer, HNSC, and BRCA (Fig. 7; Supplementary Fig. S12). Only for PAAD, we confirmed an association with DFI but not OS. We note that we have used the Pan Cancer Atlas outcome data (30) in contrary to other studies that might have used earlier or other versions of the data, potentially leading to differences in results.

Our other analyses of PAAD suggest that this association is driven by signal that stems from fibroblasts, not from cancer cell–intrinsic mesenchymality. It might be that the characteristics of some pancreatic tumors to form conglomerates of cancer cells surrounded by CAFs indeed is relevant for DFI, but not OS. This difference between DFI and OS is hard to explain without further studies. We believe that, reinforcing the statements of Venet and colleagues, single observations of differences in survival in stratification by EMT-related signatures cannot be regarded as validation of the ability of these signatures to score cancer cell–intrinsic mesenchymality/stemness characteristics (63).

Our findings show that the expression signals of genes and gene expression signatures that are proposed to be associated with EMT or stemness in cancer should be interpreted with caution when they are investigated in clinical tumor samples. If the experimental procedures are not tightly controlled, the scores of EMT-related signatures in cancer tissues are mostly driven by non-tumor cells such as fibroblasts. Fibroblast content is often inversely correlated with cancer cell content. High fractions of fibroblasts can result from the inclusion of fibroblasts from the TME or from the tumor-adjacent neighboring stroma of the tumor macroenvironment (Fig. 3). High scores of EMT-related signatures are often not the result of cancer cell–intrinsic stemness or the mesenchymality phenotype of tumor cells, but rather the result of a sampling artifact.

Consequently, we recommend implementing experimental procedures to maintain high cancer cell content in clinical gene expression profiling studies with the intent to use EMT-related gene signatures to identify cancer cell–intrinsic tumor stemness or mesenchymality.

## Supplementary Material

Supplementary Figure S1 Association of EMT-related gene expression signature scores with tumor purity and fibroblast signatures in TME naïve samples

Supplementary Figure S2 RosettaSX analysis of TCGA and CCLE breast cancer samples.

Supplementary Figure S3 RosettaSX analysis of TCGA and CCLE GBM and central nervous system cancer samples.

Supplementary Figure S4 RosettaSX analysis of TCGA and CCLE pancreas cancer samples.

Supplementary Figure S5 Correlation coefficients, comparing EMT-related gene expression signature scores with TME and non-TME signatures

Supplementary Figure S6 Influence of fibroblast cell line lineage on the coherence of EMT-related gene expression signature scores.

Supplementary Figure S7 Correlation coefficients between EMT-related gene expression signature scores and cell type abundances (Ecotyper) in breast cancer.

Supplementary Figure S8 Correlation coefficients between EMT-related gene expression signature scores and cell type abundances (Ecotyper) in head and neck squamous cell carcinomas.

Supplementary Figure S9 Correlation coefficients between EMT-related gene expression signature scores and cell type abundances (Ecotyper) in colorectal cancer.

Supplementary Figure S10 Correlation coefficients between EMT-related gene expression signature scores and cell type abundances (Ecotyper) in pancreas cancer.

Supplementary Figure S11 Percentages of differentially expressed genes in across different cell types of breast cancer, colorectal cancer, head and neck squamous cell carcinoma and glioma.

Supplementary Figure S12 Multivariate cox proportional hazard analysis association between EMT-related gene expression signature scores and disease free survival and overall survival.
