# Supplementary material for: Stromal Signals Dominate Gene Expression Signature Scores That Aim to Describe Cancer Cell–intrinsic Stemness or Mesenchymality Characteristics
Source: Cancer Res Commun. 2024 Feb 23;4(2):516–29. doi: 10.1158/2767-9764.CRC-23-0383 (PMC10885853; doi:10.1158/2767-9764.CRC-23-0383)
Supplement: Supplementary Figure S1 — Association of EMT-related gene expression signature scores with tumor purity and fibroblast signatures in TME naïve samples [file crc-23-0383-s01.docx]

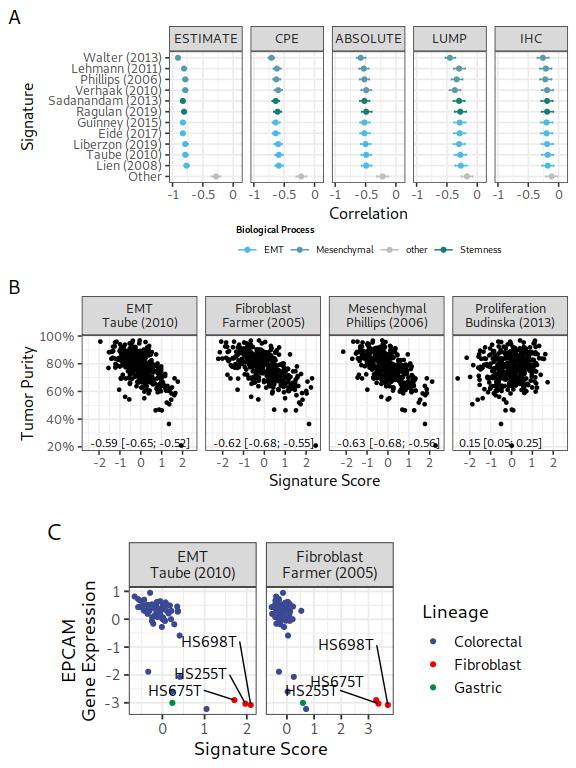


Supplementary Figure S1: Comparison of signature scores with cancer cell content in TCGA CRC and epithelial markers in TME-naïve colorectal cell lines A: Association between gene expression scores for mesenchymal, EMT, stemness signatures, and tumor purity in TCGA CRC. The ‘others’ category summarizes the average correlation, lower and upper 95th percentile of thirty-three coherent gene expression signatures. Colors indicate different biological processes, and error bars indicate the upper and lower 95th percentile confidence intervals. B: Scatter plots comparing mesenchymal, stemness, EMT, and cancer-related gene expression signatures with tumor purity. C: Scatter plot comparing an epithelial marker (EPCAM) with EMT and fibroblast signature scores in CRC cell lines, colored by cell line lineage. Top 3 cell lineswith highest EMT/Fibroblast gene expression signature scores were labeled.
