# Supplementary material for: Stromal Signals Dominate Gene Expression Signature Scores That Aim to Describe Cancer Cell–intrinsic Stemness or Mesenchymality Characteristics
Source: Cancer Res Commun. 2024 Feb 23;4(2):516–29. doi: 10.1158/2767-9764.CRC-23-0383 (PMC10885853; doi:10.1158/2767-9764.CRC-23-0383)
Supplement: Supplementary Figure S2 — RosettaSX analysis of TCGA and CCLE breast cancer samples. [file crc-23-0383-s02.docx]

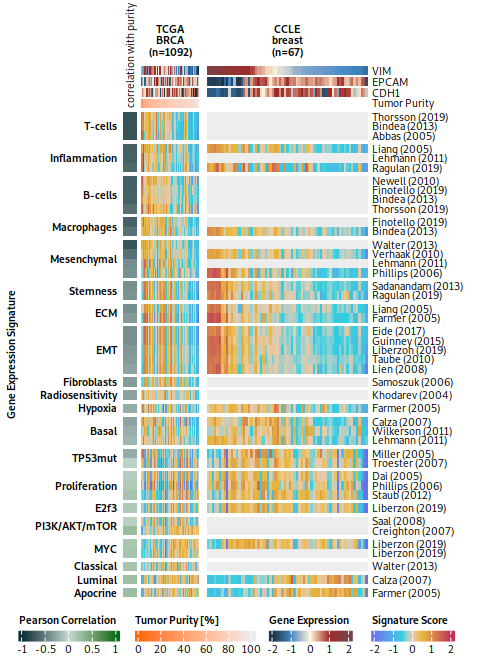


Supplementary Figure S2: RosettaSX analysis for forty-four filtered gene expression signatures in breast TCGA RNA-seq samples and DepMap breast cancer cell lines. Cell lines with elevated EMT-related signature scores have a mesenchymal origin (HS281T, HS343T, HS606T, HS578T, HS274T)
