# Supplementary material for: Stromal Signals Dominate Gene Expression Signature Scores That Aim to Describe Cancer Cell–intrinsic Stemness or Mesenchymality Characteristics
Source: Cancer Res Commun. 2024 Feb 23;4(2):516–29. doi: 10.1158/2767-9764.CRC-23-0383 (PMC10885853; doi:10.1158/2767-9764.CRC-23-0383)
Supplement: Supplementary Figure S3 — RosettaSX analysis of TCGA and CCLE GBM and central nervous system cancer samples. [file crc-23-0383-s03.docx]

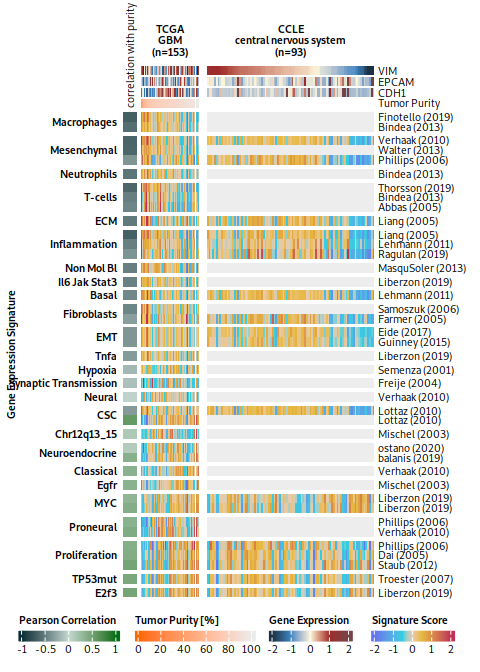


Supplementary Figure S3: RosettaSX analysis for forty filtered gene expression signatures in glioblastoma TCGA RNA-seq samples and DepMap central nervous system cell lines.
