# Supplementary material for: Stromal Signals Dominate Gene Expression Signature Scores That Aim to Describe Cancer Cell–intrinsic Stemness or Mesenchymality Characteristics
Source: Cancer Res Commun. 2024 Feb 23;4(2):516–29. doi: 10.1158/2767-9764.CRC-23-0383 (PMC10885853; doi:10.1158/2767-9764.CRC-23-0383)
Supplement: Supplementary Figure S5 — Correlation coefficients, comparing EMT-related gene expression signature scores with TME and non-TME signatures [file crc-23-0383-s05.docx]

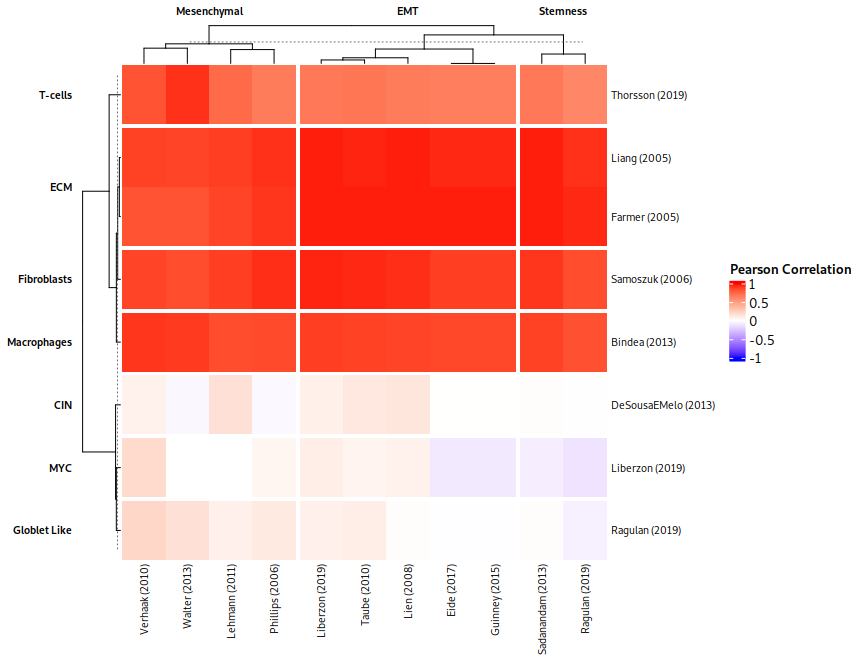


Supplementary Figure S5: Pearson correlation coefficients of EMT-related signatures and TME (T-cells, ECM, fibroblasts, macrophages) and non-TME signatures (CIN, MYC and Globlet Like)signatures in TCGA CRC.
