# Supplementary material for: Stromal Signals Dominate Gene Expression Signature Scores That Aim to Describe Cancer Cell–intrinsic Stemness or Mesenchymality Characteristics
Source: Cancer Res Commun. 2024 Feb 23;4(2):516–29. doi: 10.1158/2767-9764.CRC-23-0383 (PMC10885853; doi:10.1158/2767-9764.CRC-23-0383)
Supplement: Supplementary Figure S6 — Influence of fibroblast cell line lineage on the coherence of EMT-related gene expression signature scores. [file crc-23-0383-s06.docx]

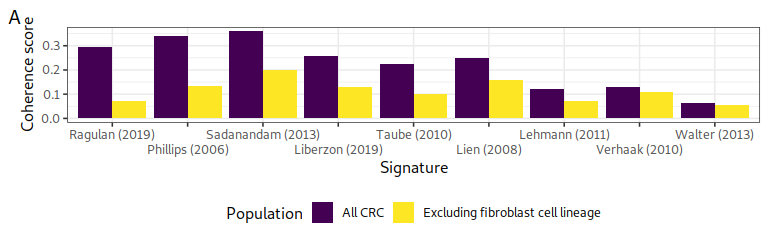


Supplementary Figure S6: Coherence score for gene expression signatures in colorectal cancer cell lines strongly affected by mesenchymal origin lines. While red bars represent the correlation coefficient for all seventy-four cell lines, the blue bars show the coefficient for the 71 cell lines (excluding lines with fibroblast lineage).
