# Supplementary material for: Stromal Signals Dominate Gene Expression Signature Scores That Aim to Describe Cancer Cell–intrinsic Stemness or Mesenchymality Characteristics
Source: Cancer Res Commun. 2024 Feb 23;4(2):516–29. doi: 10.1158/2767-9764.CRC-23-0383 (PMC10885853; doi:10.1158/2767-9764.CRC-23-0383)
Supplement: Supplementary Figure S7 — Correlation coefficients between EMT-related gene expression signature scores and cell type abundances (Ecotyper) in breast cancer. [file crc-23-0383-s07.docx]

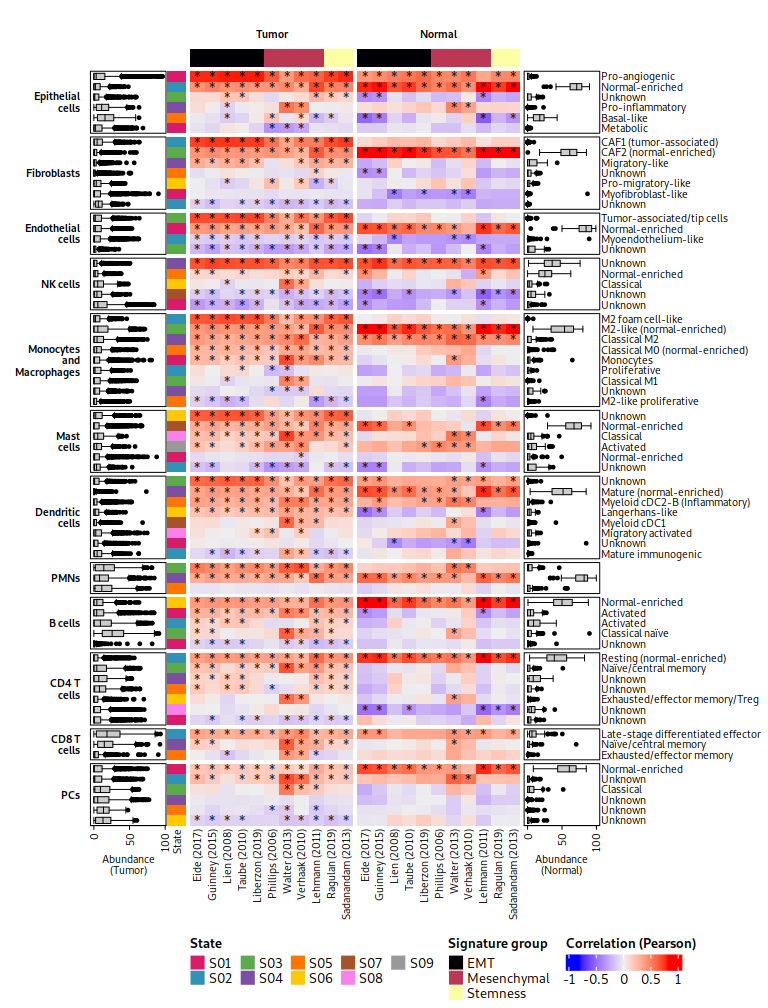


Supplementary Figure S7: Correlation of gene expression signature scores and cell type abundances in TCGA BRCA tumor and normal adjacent to the tumor tissue. Each cell displays the correlation of a gene expression signature (rows) with a specific cell type abundance (columns). Top and bottom boxplot annotations indicate the distribution of cell type abundances in BRCA samples.
