# Supplementary material for: Stromal Signals Dominate Gene Expression Signature Scores That Aim to Describe Cancer Cell–intrinsic Stemness or Mesenchymality Characteristics
Source: Cancer Res Commun. 2024 Feb 23;4(2):516–29. doi: 10.1158/2767-9764.CRC-23-0383 (PMC10885853; doi:10.1158/2767-9764.CRC-23-0383)
Supplement: Supplementary Figure S11 — Percentages of differentially expressed genes in across different cell types of breast cancer, colorectal cancer, head and neck squamous cell carcinoma and glioma. [file crc-23-0383-s11.docx]

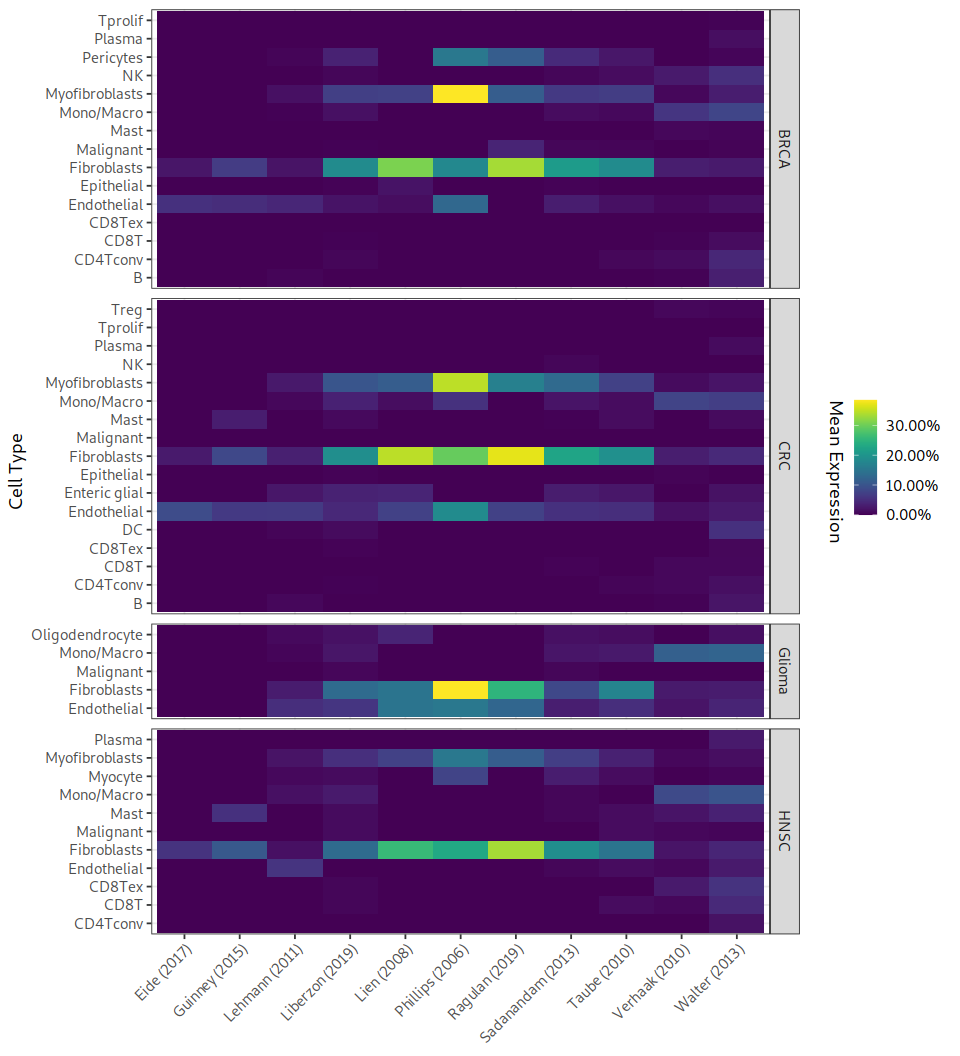


Supplementary Figure S11: Percentage of differentially expressed genes in BRCA, CRC, HNSC, and Glioma. Each cell-type was analyzed in comparison to all other cell lines within the respective dataset. Percentages were averaged across datasets for the same indication.
