# Supplementary material for: Stromal Signals Dominate Gene Expression Signature Scores That Aim to Describe Cancer Cell–intrinsic Stemness or Mesenchymality Characteristics
Source: Cancer Res Commun. 2024 Feb 23;4(2):516–29. doi: 10.1158/2767-9764.CRC-23-0383 (PMC10885853; doi:10.1158/2767-9764.CRC-23-0383)
Supplement: Supplementary Figure S12 — Multivariate cox proportional hazard analysis association between EMT-related gene expression signature scores and disease free survival and overall survival. [file crc-23-0383-s12.docx]

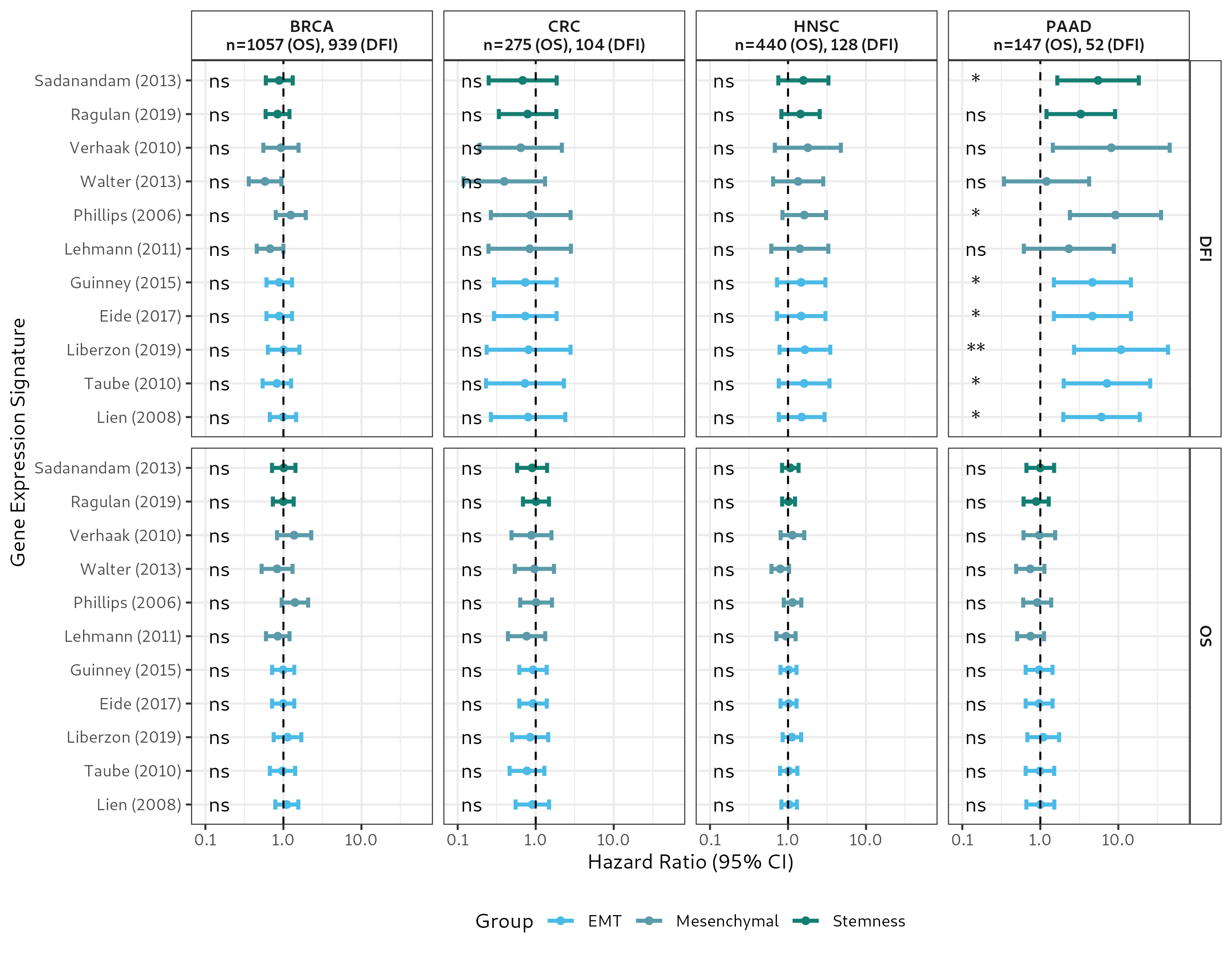


Supplementary Figure S12: Multivariate cox proportional hazards analyses for EMT-related signature scores and TCGA outcomes, adjusting for tumor stage and cancer cell content. P-values were adjusted for multiple testing within each panel using the Holm adjustment method. Upper panels: Association with DFI, excluding GBM and READ due to short follow-up or low number of events. Lower panels: Association with OS. ns: not significant, **: p < .01 *: p < .05, ns: p > .05
